# Challenges and opportunities in ECMO monitoring: A mixed-methods appraisal

**DOI:** 10.1051/ject/2026022

**Published:** 2026-09-15

**Authors:** Wei Yin, Alisa Permessur, George Pittas, Shiffoni Sukhlal, Nicholas Chow, Steven Pinheiro, Andrew Cobos, Giancarlo Sabetta, Jan Cahill, Saleem Zaidi, Jon Longtin, Jonathon Schwartz

**Affiliations:** 1 Department of Biomedical Engineering, Stony Brook University Stony Brook NY 11794 USA; 2 Department of Physiology and Biophysics, Stony Brook University Stony Brook NY 11794 USA; 3 Renaissance School of Medicine, Stony Brook University Stony Brook NY 11794 USA; 4 Cardiovascular Perfusion Division, Department of Cardiac Surgery, Renaissance School of Medicine, Stony Brook University Stony Brook NY 11794 USA; 5 Department of Anesthesiology, Renaissance School of Medicine, Stony Brook University Stony Brook NY 11794 USA; 6 Department of Mechanical Engineering, Stony Brook University Stony Brook NY 11794 USA

**Keywords:** ECMO, Blood coagulation, Bioengineering, Database, Device

## Abstract

Extracorporeal membrane oxygenation (ECMO) provides heart/lung support for critically ill patients. While advances in research have improved the overall design and performance of ECMO circuits, complications such as hemolysis and thrombosis persist, and the dysfunction of the ECMO machine itself can pose significant risk to patients. Many parameters need to be closely monitored during an ECMO run, and it is often labor and resource-intensive. Currently, there are few commercially available automated ECMO circuit monitoring systems that can provide continuous, holistic monitoring for ECMO circuit health and provide real-time clinical recommendations.

This mixed-methods review (through literature review and customer discovery interviews) discussed key parameters relevant to ECMO circuits and monitoring, including bleeding, thrombosis, hemolysis, flow and pressure conditions, and gas exchange. Their importance in ECMO monitoring, current practices, and available technologies were highlighted. In addition, a customer discovery study, supported by the New York regional I-Corps program, was conducted to identify the “pain points” in ECMO circuit monitoring through interviews with healthcare professionals and other stakeholders experienced in ECMO care/use.

Findings from both the literature review and the customer discovery study indicated that ECMO monitoring and management require significant resources, due to the variability and complexity of patients’ conditions, challenges in collecting patient and ECMO circuit data, and the need for a comprehensive understanding of the entire patient-ECMO ecosystem. Improvements in understanding complex ECMO data, incorporating new sensors into the monitoring system, and establishing comprehensive ECMO data pipelines represent key steps towards developing “smart” ECMO monitoring that can serve as a clinical decision support system.

## Introduction

*Extracorporeal membrane oxygenation* (*ECMO*) has become a widespread tool for life support in critically ill patients. Based on the Extracorporeal Life Support Organization (ELSO) registry, over 148,000 adults, 41,000 children, and 51,000 neonates had received ECMO support worldwide as of January, 2025 [1]. These values likely underestimate the total ECMO cases since the first clinical reports of its use in the 1970’s [2, 3].

The major components of an ECMO circuit include a mechanical blood pump, circuit tubing, access/return cannulas, and a membrane lung (oxygenator). The mechanical blood pump is most often a centrifugal pump, whose speed (RPM) is controlled by an ECMO specialist, such as a perfusionist, to maintain a desired blood flow rate. The circuit tubing usually consists of polyvinyl chloride, sometimes coated with anticoagulants [4, 5]. ECMO can be used for heart and lung support (venoarterial, or VA) or only lung support (venovenous, or VV) [6–10]. Several vendors provide commercial membrane oxygenators used in ECMO circuits, including the Abbott Eurosets, Maquet QUADROX, Sorin EOS, and Medtronic Nautilus, etc. [11].

Both basic science and clinical research have significantly improved the design and performance of ECMO circuits. Patient and circuit complications, such as intravascular hemolysis, systemic inflammation, platelet destruction, bleeding, and thromboembolism, however, remain unresolved [10]. The dysfunction of the ECMO machine itself could be detrimental as well, as failure to provide adequate cardiac support (flow failure) or lung support (gas exchange failure) can cause irreversible patient injury.

All ECMO machines have modular or integrated console displays that report pressure, flow rate, among other variables (e.g., oximetry readings and estimated hemoglobin) in real-time. Historically, this data was typically stored on local machines (i.e., the ECMO console) and rarely transmitted to remote or cloud storage. This changed only recently with the emergence of connected care platforms, which now enable data transfers at regular time intervals [12].

However, readings of flow and pressure data from the console are typically recorded only once or twice an hour (manually) into the patient’s electronic health record [13, 14]. Data security has become a critical aspect to consider among the varied potential data pipelines and repositories that could be utilized. Utilizing offsite cloud solutions for data management may introduce risks, even beyond cybersecurity, like latency, interoperability, data quality and loss with transfers, data ownership and governance, dependence on external vendors, and regional or country-wide regulations beyond patient privacy.

Furthermore, lab tests such as coagulation status [Activated Clotting Time (ACT) or Partial Thromboplastin Time (PTT)], blood gases, and blood cell counts are typically obtained on the order of several hours, and on an ad-hoc basis. Data on ECMO flow conditions and patients’ lab results are critical to support clinicians’ decision-making in anticoagulant administration, neurological complication prevention, and overall risk mitigation [15]. An automated, supervisory system capable of capturing continuous, holistic ECMO data such as flow, pressure, gas exchanges, clotting, hemolysis, etc., coupled with real-time data analysis to detect early trends or anomalies, i.e., a clinical decision support system (CDSS) [16], is highly desirable to reduce/prevent ECMO circuit dysfunction to improve patient care.

There is a wide variability in monitoring capability in ECMO circuit components. Automated systems have been used to provide real-time monitoring of laboratory tests and patients’ vital signs, send alerts, and provide clinical recommendations [17]. The advantages of a CDSS for ECMO monitoring include resistance to fatigue-related errors, reduced staffing burden, and earlier detection of abnormal conditions. However, challenges include i) alteration of clinical workflow causing skill atrophy and mode confusion [18]; ii) clinician dependence on an automated system may develop automation bias [19]; and iii) training requirements for all clinical users. Success is also limited by the number of monitored parameters and the system’s predictive capability beyond threshold reporting. So far, it is not conclusive whether the current CDSS could effectively improve patient outcomes [12].

A "smart" monitoring system - one that synthesizes continuous ECMO data with machine learning-based trend detection to provide early warning and clinical decision support, is therefore highly desirable [20] and could extend ECMO care capacity in low-resource settings [21, 22].

Currently, there are few commercially available automated ECMO circuit monitoring systems or Electronic Medical Record (EMR)-based systems that provide continuous, smart, and holistic monitoring of ECMO circuit health with real-time clinical recommendations. An example is the Maquet Cardiohelp system, which can provide autoregulation by incorporating a pressure-based pump protection approach. It continuously monitors venous inlet pressure and can automatically reduce blood pump RPM if excessive negative pressure is detected. Once the negative pressure stabilizes at the desired level, the system will restore the pump RPMs to their prior operating level [23]. Important questions thus arise: is such a system needed to improve ECMO care outcomes, and at what cost? If a “smart” ECMO monitoring system were available, what information would be collected, analyzed, and reported? This mixed-methods review addresses these questions through a literature review of ECMO performance monitoring and a customer discovery study examining current practice and clinician perspectives on an automated monitoring system. Many prior reviews have thoroughly examined variables that are typically collected for ECMO monitoring [20, 24]. In this review, we investigated monitoring practices from both the literature and user perspectives, focusing on pre-existing, real-world challenges to address in pursuit of a smart CDSS system.

## Methods

### Literature search

A literature search was conducted on PubMed and Google Scholar. Keywords for the search included ECMO, cardiorespiratory failure, extracorporeal life support, hemorrhage, oxygenator, thrombosis, clotting, system exchange, hemolysis, neonatal and pediatric ECMO, gas exchange, and ECMO flow and pressure. Articles were reviewed without restriction on date or publication type by a team of biomedical engineers and students, critical care physicians, perfusionists, and medical students. No formal inclusion/exclusion criteria or quality assessment framework was applied. Relevant studies were organized thematically according to ECMO circuit component, complications, and monitoring approaches, and synthesized through iterative discussions among the team members.

### Customer discovery

To better understand the clinical and implementation challenges associated with ECMO circuit monitoring, we performed a customer discovery study with ECMO users and other stakeholders.

Through a National Science Foundation regional I-Corps program [25] (Gotham Innovation Gambit, Fall 2023), 45 stakeholders in the United States and Canada were interviewed between September and October 2023 by biomedical engineering students regarding their experience with ECMO, especially their perceived pain points in workflow, patient management, clotting risk, and system failure management. The goal of the customer discovery study was to understand if there was a need to improve current ECMO circuit monitoring approaches and their potential economic and societal impact. Table 1 summarizes the eligibility criteria for interviewees included in the customer discovery study.

**Table 1:** Customer discovery eligibility criteria.

Target clinicians & professionals	Medical professionals who use ECMO, including perfusionists, nurses, doctors, and respiratory therapists.
	Academic researchers and scientists.
	ECMO specialists and support engineers.
	Sales and hospital administrators.
Intervention	Direct involvement with VV and VA ECMO use for both adults and neonates.
Geographic Location	United States and Canada
Institutional diversity	Academic universities, university hospitals, community medical centers, different hospital systems, and smaller medical centers.
Outreach	Professional networking platforms such as LinkedIn; in-person conferences and interviews; ECMO organizations; emails to eligible participants.
Method of interviews and data collection	In person, video (or Zoom) conferences, phone calls.
	Questionnaires were documented.
Outcomes	Perceived pain points in workflow, adverse effects in patients, clotting, and ECMO system failures.

Candidate clinicians and professionals (“customers”) were identified through web searches (~50–60%) and referrals from successfully contacted interviewees. Preference was placed on the northeast US or Canadian regions due to various constraints. The most significant challenge was providers’ availability, as most clinical providers were only accessible during evenings, breaks, or while on call. Frequent scheduling conflicts, last-minute cancellations, no-shows, and short interview windows further limited the team’s ability to conduct deeper conversations. Limited public access to providers’ emails and direct contact numbers also made it difficult to communicate. Overall, we contacted approximately 100 clinicians and professionals and were successful with 45 interviews. The interviews were conducted virtually or in person. Participation in the interview was voluntary without reimbursement from the interviewers. A structured questionnaire was used to guide the interview (see Supplementary Document for Interview Questionnaires), along with unplanned questions and discussion. Interviews typically lasted no more than 45 min per interviewee.

## Results

### Literature review on ECMO monitoring – current approaches

The following sections discuss ECMO circuit monitoring, circuit-related complications, and parameters commonly used to evaluate ECMO circuit health.

### Bleeding and thrombosis monitoring

Bleeding is a common complication for ECMO patients. A 2022 single-center retrospective study reported that among 105 ECMO patients evaluated, major bleeding occurred in 31% of the patients and was associated with higher in-hospital mortality [26]. Many bleeding events occurred at cannula insertion sites, surgical incisions, and the oronasal cavity, while a lower proportion occurred in the gastrointestinal and urinary tracts [26, 27]. Hvas et al. reported that of 100 patients, 66 experienced at least one bleeding incident while on ECMO, with 76% occurring on day 1 or 2, and the rest on day 3 or later [28]. They concluded that there was no significant difference in bleeding incidence between VA and VV ECMO. Similar observations were reported by others. Kalbhenn and Zieger reported that 30–60% of patients on VV-ECMO had bleeding complications [29]. Ellouze et al. reported that among VA-ECMO patients, about 46% had major bleeding events [30]. A systematic review on VV ECMO stated that the rate of any bleeding could reach 29% [31], with a 10% risk of major bleeding [32]. Bleeding may be caused by various reasons, such as cannulation [33, 34], excessive anticoagulation, platelet dysfunction, insufficient coagulation factors, or fibrinolysis [10].

To address bleeding complications, researchers have investigated the feasibility of withholding anticoagulants in ECMO patients. Olson et al. screened 443 publications on ECMO studies and reported that about 201 patients had received anticoagulation-free ECMO treatment (about evenly distributed between VV- and VA-ECMO). Their report concluded that the median run time for anticoagulation-free ECMO was 4.75 days, and the major reasons for the anticoagulant-free approach were thrombocytopenia and hemorrhage. Biocompatible ECMO tubing was used in more than 50% of these studies, and some patients received antiplatelet medications [35]. However, thrombosis occurred in more than 10% of the cases, mostly within the membrane lungs.

Thrombosis inside the ECMO circuit or in patients is a serious complication. How the ECMO circuit affects the coagulation system and the inflammatory pathways has been extensively studied [27, 36–38]. Still, thrombosis remains one of the main reasons for membrane lung dysfunction and system exchange [39]. Recent research has shown that red-blood-cell-rich or fibrin-rich thrombi can form anywhere within the ECMO circuit (e.g. the membrane lung, the tubing, the pump, etc.) [40]. Thrombus buildup in the membrane lung can cause ECMO circuit dysfunction [40–42]. Depending on ECMO circuit type and patient conditions, up to 46% of adult patients could develop non-device, clotting-related complications such as ischemic stroke or ventricular thrombus during the course of ECMO [43]. Up to 37% of neonatal and pediatric patients were reported to suffer from thrombotic complications, including circuit thrombosis and cerebral infarction [44]. Systemic anticoagulation has been the mainstay for preventing device-related thrombotic complications. Commonly used anticoagulants include heparin and direct thrombin inhibitors such as bivalirudin and argatroban [45, 46]. Warfarin has also been used in clinical practice, but not as commonly [43]. Anticoagulant-free ECMO has been investigated in multiple studies [47–50]. However, due to the subtle balance between clotting and bleeding, inconsistent results were reported, and this approach remains controversial [46, 51].

It is well recognized that anticoagulation during ECMO is a dynamic process that can be unpredictable and often interrupted by bleeding [52]. Biochemical assays commonly used to evaluate ECMO patient bleeding or thrombosis risk include the Activated Partial Thromboplastin Time (APTT), the Activated Clotting Time (ACT), anti-Xa level (also known as heparin activity level or HAL), and Prothrombin Time with International Normalized Ratio [53]. The APTT assay measures the time it takes for calcium-free plasma to form clots with the presence of fibrin-activating reagents, and calcium is routinely used to measure heparin and bivalirudin effects [43, 54]. The normal range of APTT measurements (20–40 s in adults) is relatively large and heavily dependent on instruments and/or reagents used (60–90 s could be acceptable) [55, 56]. The ACT assay measures clot formation time in whole blood upon contact activation. A range of 140–240 s in ACT measurements is often considered acceptable, but there is no standardized normal range for clinical evaluation [57]. The growing use of bivalirudin for ECMO anticoagulation has triggered awareness and interest in utilizing higher-specificity assays such as the ecarin chromogenic assay and dilute thrombin time [58].

The D-dimer assay, which measures the concentration of D-dimer (the cleaved product of fibrinolysis) in blood, can be used to measure thrombus formation and its subsequent breakdown and to evaluate the potential of thrombus formation within the membrane lung [59]. Unlike APTT and ACT, which directly measure coagulability, the D-dimer assay is an indirect method that detects the presence of active clots in the patient or the circuit.

Methods that measure changes in blood’s physical properties have also been explored for coagulation monitoring. Examples include thromboelastography and rotational thromboelastometry. These methods measure the viscoelasticity of whole blood, which changes during clot formation and clot lysis [60, 61]. These methods have been used to support liver transplant and other noncardiac surgeries, assess the clotting risks in end-stage liver disease and sepsis [62–66], and monitor anticoagulation in ECMO and LVAD (left ventricular assist device) patients [67, 68]. However, due to the lack of strong evidence to support their overall effectiveness, adoption of their use clinically has been slow, although suggested as an option for monitoring by ELSO [69–71].

In summary, it is critical to monitor both bleeding and clotting simultaneously during ECMO use. Many anticoagulants have a narrow therapeutic index [72], and overdosing can lead to life-threatening bleeding, whereas subtherapeutic dosing can lead to clot formation in the circuit or the patient. Bleeding and clotting are highly dynamic processes that need to be carefully managed via frequent lab testing. However, due to large variations in ECMO circuit equipment/configurations and patient conditions, a single reproducible strategy for therapeutic anticoagulation may not be possible. As such, ELSO guidelines advocate for a tailored anticoagulation monitoring approach centered around clinical judgment [70]. Furthermore, current methods rely on intermittent blood sampling for laboratory assays, which risks exposing the patient and the circuit to over- and under-dosing of anticoagulants. The continuous and holistic monitoring of bleeding and thrombotic events across the ECMO circuit and within the patient is urgently needed.

### Hemolysis monitoring

Hemolysis is defined as the destruction of red blood cells and the release of hemoglobin into the surrounding plasma. In ECMO, hemolysis is commonly monitored using plasma-free hemoglobin (PFHb), where severe hemolysis is often described as PFHb greater than 50 mg/dL [10, 73, 74]. This complication can worsen anemia, increase blood transfusion use, cause kidney injury [75–77], and forewarn pump or membrane lungs thrombosis. Therefore, real-time monitoring of hemolysis within the ECMO circuit and in the patient is desirable [74]. It was reported that hemolysis occurred in about 18% of ECMO patients and could lead to sepsis and increased mortality [78]. A retrospective study with 1,063 patients demonstrated that VA ECMO patients had a higher rate of severe hemolysis compared to VV ECMO patients [79], mostly due to patients’ conditions rather than the ECMO circuit itself. The same study concluded that independent of ECMO mode, neither the membrane lung model, pump model, nor cannulation sizing and strategy were directly associated with hemolysis severity. However, this conclusion differed from others’ findings. In the review by Dufour et al. on ECMO hemolysis, it was stated that thrombosis, excessive mechanical stress, pump rotation speed, cavitation, and blood-air interface within the ECMO circuit can all lead to hemolysis [80].

In the cardiovascular system, blood flow-induced shear stress usually ranges between 1 and 10 Pa. However, it can increase dramatically in the ECMO circuit [73]. For example, within the blood pump, elevated shear stress over 50 Pa has been observed, which can cause hemolysis [81, 82]. Due to the high velocity as well as the use of small-diameter cannulas, turbulent flow can develop within the blood pump, leading to large deformation and rupture of red blood cells, and increasing the risk of hemolysis. Hemolysis can also be caused by the large pressure gradient across the membrane lung. Previous studies have shown a potential correlation between the pressure drops across the membrane lung and the *Normalized Index of Hemolysis for Oxygenators* (NIHO) [83, 84], an index that describes the severity of hemolysis caused directly by the membrane lung, when the blood flow rate was accounted for. However, due to the variations in the models of membrane lung and blood pumps used in ECMO circuits, the occurrence of hemolysis is often circuit-specific.

As red blood cells are damaged or ruptured, hemoglobin is released into plasma, forming what is known as free hemoglobin (fHb) or PFHb. Free hemoglobin can be detrimental to the body as its build-up can cause a decline in tissue oxygen levels and the generation of degradation and crosslinking products with cytotoxic and inflammatory activities [85–87]. fHb has also been shown to decrease nitric oxide concentration within the blood, which can cause vasoconstriction and platelet activation [73, 88]. However, there is no standardized testing for hemolysis. Instead, select biomarkers are measured to estimate the severity of red blood cell damage, or hemolysis. The most prominent hemolysis biomarker is fHb itself. fHb has been shown to be a key predictor for patient mortality as well as clinically significant events regarding the ECMO use, including potential circuit swaps [73, 89–91]. Rapid rises in PFHb or extreme values could also signal pump thrombosis. Testing of fHb is a readily available lab test, which is recommended to take place once every 12 h or at the physician’s request for ECMO patients, in order to monitor and manage the hemolysis risk [92].

Other clinical biomarkers for hemolysis include lactate dehydrogenase (LDH), bilirubin, haptoglobin, and aspartate transaminase [73], carboxyhemoglobin [93], and H-index [94, 95]. Changes in these markers have been reported to correspond to increases in plasma free hemoglobin but may lack specificity. Thus, measurements of these biomarkers can offer some supplementary information on hemolysis when fHb testing is not available. However, like fHb, all of these biomarkers require laboratory testing, which consumes staffing resources, patients’ blood mass, and delays clinicians’ decision-making due to the intermittent sampling inherent in the testing design [92].

Alternative approaches not requiring lab testing have been investigated to gauge hemolysis in ECMO circuits. Burger et al. evaluated the predictive potential of ECMO blood flow rate as an indicator of hemolysis for patients with acute respiratory distress syndrome (ARDS). Using a linear regression model, they demonstrated that for ARDS patients on VV ECMO, elevated overall ECMO flow rate was associated with increased fHb, hemolysis, and poorer patient outcome [96]. However, it was not clear whether the increased flow rate/fHb resulted from an elevated pump speed, or whether other processes or modifiable factors, such as anticoagulation, were considered. In contrast, both in vitro and in silico models demonstrated that increased hemolysis was only associated with low flow rate in centrifugal blood pumps used in ECMO [97, 98].

In summary, as a well-accepted biomarker for hemolysis, fHb is monitored during ECMO care. However, fHb measurements require lab testing intermittently, and the results are usually not immediately available. Alternative methods have been investigated to detect hemolysis without lab testing. However, results obtained from different studies and models were inconsistent with the conclusions reached, thereby limiting immediate adoption into clinical practice. Development of continuous, automated hemolysis detection technologies that can meaningfully alter clinical management decisions and improve outcomes remains an important opportunity for innovation.

### Flow and pressure monitoring

It is important to monitor flow and pressure parameters of the ECMO circuit to ensure adequate patient life support and safety [99]. Low flow, particularly with inadequate anticoagulation or stagnant flow fields, could increase the risk of thrombosis. In silico modeling coupled with in vitro studies suggests that high pump speeds and flows could induce hemolysis, although direct clinical evidence remains limited beyond several retrospective studies [3, 100]. Changes in ECMO circuit flow may also indicate cannula malposition, thrombotic obstruction, or tubing kinks [101]. Pressure fluctuations at different locations within the ECMO circuit may reflect changes in cannula position, vascular resistance, or membrane lung problem [24]. In some cases, extreme changes in circuit flows and pressures serve as alarms for leaks or an elevated risk of circuit rupture.

Membrane lung (ML) flow conditions are closely monitored. Since the pump in the ECMO circuit is designed to maintain a consistent target speed and flow rate [12], it is a common practice to monitor the pressure drop (ΔP) across the ML. An increase in pressure drop often indicates an increase in ML resistance, which is often caused by thrombosis, fibrin, or cellular deposition within the ML [20, 102–105]. Consequently, thrombosis and cellular deposition may be further exacerbated inside ML as resistance increases. The overall ML resistance can be calculated as the ratio of ML pressure drop over blood flow rate (ΔP/Q). It was reported that ML resistance could rise by 2 to 4-fold when acute ML thrombosis occurs [106]. ML can gradually lose efficiency as ECMO run time increases. Consequently, the ML would need to be replaced, which is often referred to as a system exchange [107], circuit exchange [108], or oxygenator exchange [109]. A study by Lubnow et al. reported that among 265 patients on ECMO support, 83 (31%) required system exchange. Among those, significant increases in ML pressure drop were observed in 26 patients, 16 with acute clot formation in the ML and 10 with progressive clot formation in the ML [106]. A retrospective study on 45-ECMO patients by van Minnen et al. reported 29 ML exchanges in 19 patients (42%); and the changed ML pressure drop had been identified as one of the key parameters associated with system exchange [110]. ELSO provides general guidelines regarding pressure and flow monitoring [111]. Flow rate and pressure drop across the ML are routinely monitored and often recorded manually by the clinical team, including ECMO specialists and perfusionists, as indicators for potential or imminent ECMO circuit dysfunction. However, the frequency of recording and structure of documentation vary across centers. Since pressure and flow can vary largely depending on the cannulas, ML model, pump model, patient blood volume, and systemic vascular resistance, there are no exact target thresholds to refer to [24], making troubleshooting or decision-making challenging sometimes. When the blood pump speed was stable, a change in ECMO flow rate may be caused by preload or afterload problems. Preload problems may include hypovolemia, bleeding, venous drainage, cannula obstruction, venous tubing kinks, or venous-side thrombosis. Afterload problems may include ML thrombosis, elevated systemic vascular resistance, arterial return cannula obstruction, arterial tubing kinks, or arterial-side circuit thrombosis [112]. Hypovolemia may cause chattering, another circuit-related complication that can exacerbate hemolysis [113]. In general, ML pressure drops should increase with blood flow rate, regardless of the ML type [114]. However, across different MLs, target values for ML pressure drop can vary significantly due to variability in ML geometry, size, and materials. For example, the target pressure drop for the Maquet system ranged between 6 and 34 mmHg, and that for Euroset’s AMG PMP (distributed by Abbott) system could go up to 250 mmHg [115].

Sarathy et al. proposed a shunt system within the ECMO circuit to achieve continuous and automatic monitoring of ML obstruction [105]. Using both in vitro and mathematical models, they demonstrated that shunt flow increased as ML obstruction worsened. Using a similar setup, Badheka et al. suggested that shunt flow can be used as a simple yet sensitive marker (in addition to pressure drop) to monitor ECMO complications, including ML obstruction, cannula obstruction, and elevated systemic vascular resistance [102]. However, there is no standard protocol for interpreting shunt flow changes during ECMO circuit monitoring, and more studies and clinical trials are needed to validate this approach and the impact of ML geometry on shunt behavior. Furthermore, shunt-based monitoring protocols would need to account for institutional variations in the pump and ML that comprise the circuit.

The use of ML pressure as a marker for ECMO circuit health monitoring also remains controversial, with some questioning its effectiveness and reliability. Zakhary et al. noted that ML resistance might be more specific than pressure gradients, and that a universal pressure level cutoff is not feasible due to variations in resistances by ML geometry [116]. A case study reported by Wahl et al. suggested that increased ML pressure should not be the only marker for monitoring ML dysfunction. In that study, despite unchanged pump speed, both the ML flow rate and pressure drop decreased steadily, due to a large clot blocking one of the ML outlets [117]. Oxygenator blood volume (OXBV) has been proposed as a complementary marker [118, 119], but its intermittent measurement technique and limited cross-model validation preclude routine clinical use at this time [114, 115]. Kaesler et al. conducted a series of in vitro experiments to determine the effect of clot formation on ML pressure drop [120]. They found that ML pressure drops only increased moderately with clot size when the total clot volume was small. They concluded that during the early stages of a clotting event, the pressure drop might only vaguely indicate ML obstruction; however, at a later or more advanced stage of clot formation, the pressure drop could become more reliable. Therefore, ML pressure drop should be analyzed alongside other indicators, including blood flow, oxygen transfer, post-ML oxygenation, and carbon dioxide clearance. ML failure may be seen as an excessive pressure drop above the safe operating range, inadequate oxygen transfer, or inadequate carbon dioxide removal. It is also important to note that even when a ML performs within the manufacturer's guidelines, it may fail to meet oxygen/carbon dioxide requirements for a patient.

While this review focuses on adult ECMO, monitoring thresholds differ substantially in pediatric and neonatal populations, given the wide range of patient sizes and flow requirements [121].

In summary, ML flow and pressure drop are recognized as important mechanical parameters to monitor during ECMO use. Normal ranges can vary greatly among different MLs and between adult and pediatric patients. There is a consensus that ML flow rate should be kept stable, and changes, especially increases, in ML pressure drop could be an alarming sign of ML obstruction (often caused by thrombosis). However, a decrease in the flow rate and pressure gradient could occasionally indicate ML obstruction. Recording ML flow rate and pressure drop into an electronic health record is often done manually at intervals, which are usually not always coordinated with blood lab testing (such as APTT or fHb). Recently, commercial solutions for high-frequency, automated data capture have started to emerge (such as Talis Perfusion with +ACG from *Getinge,* EPIC EMR/EHR*,* and Quantum Informatics −24 from *Spectrum Medical*). However, the larger challenge is linking the ECMO console data, laboratory values, and electronic health records to reduce reliance on manual data entry.

### Gas exchange monitoring

Oxygen supply and sweep gas flowmeters are a key part of the ECMO circuit [122]. The fraction of oxygen delivered is controlled through a gas blender where pure oxygen and medical air are mixed [123]. The sweep gas flow rate controls the amount of carbon dioxide removed from the blood. In the ML, oxygen uptake and carbon dioxide elimination occur simultaneously across the membrane. Pre- and post-ML blood gas analysis is critical in monitoring the gas-exchange efficiency of the ECMO circuit. Previous studies have shown that O_2_ transfer was dependent on both blood flow rate of the ML and the oxygen gradient (across the membrane), while CO_2_ elimination was dependent on gas flow rate and CO_2_ concentration at the sweep gas outlet [20]. Measurement of O_2_ and CO_2_ concentrations within the sweep gas helps to determine O_2_ uptake and CO_2_ clearance by the membrane lung [124, 125] and is a capability provided in some devices (such as Medtronic’s Nautilus™ Smart ECMO Oxygenator). Noninvasive measurement of O_2_ and CO_2_ concentrations is available through some device manufacturers [126]. However, many MLs lack integrated sensors for post-ML O_2_ partial pressure and O_2_ saturation measurements.

Arterial blood gas analysis is routinely obtained from patients at regular intervals or as needed [127]. For patients on VA ECMO, pre-ML, or venous oxygen saturation, it can effectively indicate the adequacy of oxygen delivery by the ECMO circuit [128]. Low venous oxygen saturation may indicate inadequate oxygen delivery to the patient [129, 130], which could indicate a need for further assessment of ML health. For patients on ECMO, frequent laboratory testing for arterial blood gases is often needed to support adjustments to sweep gas flows and/or overall ECMO blood flow [122]. Continuous noninvasive blood gas measurement solutions have been developed to decrease the need for blood sampling, but their use in ECMO remains limited due to sensor requirements, calibration, and regulatory status. Examples of these developments include the HYLA™ Blood Monitoring System from *Inspira* (not FDA-approved) and the CDI® Blood Parameter Monitoring system 550 from *Terumo Cardiovascular* (not widely used for ECMO practices).

Innovative ways to monitor ML gas exchange efficiency have been explored. Zakhary et al. developed an algorithmic approach to monitor ML gas exchange by evaluating ML O_2_ uptake, O_2_ partial pressure post-ML, blood flow rate, the fraction of delivered oxygen in the sweep gas, and CO_2_ partial pressure post-ML [116]. Epis and Belliato reported using a real-time continuous monitoring system (with two oximetry probes and one ultrasound flow meter) to record/trend O_2_ transport and consumption, venous/arterial oxygenation, ECMO blood flow rate, and ML pressure, to evaluate the gas-exchange efficiency of the ECMO circuit in ICU [131]. The system also evaluated CO_2_ exchange using post-ML blood gas measurement and sweep gas exhaust CO_2_ concentration with infrared spectroscopy. However, neither approach was widely adopted due to the complexity of the modeling system, the inclusion of additional sensors, and the potential interruption of the typical ECMO care workflow.

To fully assess the complex scenarios unique to each individual patient, ELSO provides rather comprehensive guidelines for ECMO gas exchange monitoring, and the key recommendations include i) continual monitoring of a patient’s arterial and venous blood gas (O_2_ and CO_2_), ii) monitor pre- and post-ML blood gas analysis daily, iii) regularly check the ML’s pressure gradient; iv) monitor ML’s blood flow rate; v) monitor and maintain adequate hemoglobin levels; vi) monitor lactate levels as an indicator of tissue perfusion and oxygenation [99, 132]. These guidelines appear feasible to implement in hospitals across a wide variety of resource settings.

In summary, gas exchange monitoring in ECMO is multidimensional, requiring simultaneous assessment of blood flow, sweep gas parameters, blood gas analysis, and hemoglobin levels. Despite the ELSO guidelines providing a practical framework, no unified protocol exists specifying how these parameters should be integrated or recorded to characterize overall circuit gas-exchange efficiency, likely because ECMO patients vary widely in condition, anticoagulation needs, and risk of bleeding/thrombosis.

Overall, bleeding, clotting, hemolysis, hemodynamic conditions of the whole ECMO circuit and the ML, and the gas exchange efficiency of the circuit, all require close monitoring to ensure the optimal performance of the ECMO-patient system. Furthermore, underlying patient conditions can highly influence the coagulation state of blood and the hemodynamic context of the ECMO circuit. Consequently, none of these parameters is completely independent of the others and should not be monitored in isolation, especially in isolation from measurements of a patient’s native lung or cardiovascular function. A team of professionals with continuous and direct communication is required to achieve successful ECMO monitoring and management with a human-in-the-loop approach [133].

### Customer discovery of ECMO monitoring challenges

Supported by a New York regional I-Corps Program [134] (Gotham Innovation Gambit, fall 2023), a customer discovery study was conducted by two senior undergraduate students and one graduate student. A total of 45 healthcare staff/professionals/industry personnel (Table 2) were interviewed (the number of interviews was recommended by the Program) and shared their experience, highlighting critical challenges in ECMO monitoring that require improvements. About 58% of the interviewees were from New York State, primarily in Stony Brook, Queens, New York City, or nearby areas on Long Island. The rest of the interviewees were from Florida, Maryland, Tennessee, Arizona, Michigan, California, and Idaho. One interviewee was from Canada. Table 3 summarizes the common pain points frequently encountered by healthcare professionals that can impact patient outcomes and the workload of medical staff.

**Table 2:** Stakeholders interviewed during customer discovery.

Professionals	Number
Nurse	7
Perfusionist	3
Physician	3
Surgeon	4
Respiratory therapist	3
Scientist	5
Biomedical engineer	5
Sales and business	15
Total	45

**Table 3:** Pain points identified through the customer discovery interviews.

ECMO complications	Pain points	Frequency of mentioning & conclusions
Bleeding	- Bleeding is a major concern for patients, especially those undergoing open-heart surgery. - Neonates are born with less than 50% of adult clotting factors, making them highly susceptible.	- This issue was frequently mentioned, highlighting the need to monitor hemoglobin trends over time for a comprehensive view of bleeding risks. - Clinicians expressed frustration with the frequent blood tests (e.g., PTT) required and the lack of automation, which disrupt workflow and strain clinical teams.
Clotting & thrombosis	- Managing thrombosis and clotting is challenging due to variable anticoagulation responses and inadequate circuit designs.	- This was an urgent need due to the lack of effective early detection methods. - Regular maintenance is crucial to prevent complications.
Hemolysis and debris formation	- Imposing significant risks for patients. - Concerns over debris accumulation in vital organs such as the liver and lungs.	- There was an urgent need for early detection to quantify and monitor debris formation, which can help manage hemolysis and reduce the risk of complications from circulating debris.
Gas control and ML issues	- Tests are slow and may not provide enough real-time information to prevent complications.	- This was frequently mentioned. - There is an urgent need for new MLs that incorporate real-time monitoring of oxygen levels and pressure for more reliable detection when a system replacement is necessary.
Flow and pressure monitoring	- Essential to prevent mechanical failures and clot formation. - The need for advanced sensors and improved integration with electronic medical records (EMR) for better documentation and monitoring.	- This was an urgent need. - Quick response times are vital when managing emergencies such as ML failures or acute blood clotting events.
Infection and sepsis	- Infection, especially sepsis, poses a significant risk for patients with large cannulas. - Symptoms are often masked by the ECMO system, leading to an increased mortality risk, particularly within 24–48 h of decannulation.	- Healthcare providers often manage fevers by adjusting patient temperatures and administering antibiotics only when necessary. - One provider suggested that advanced real-time bacterial sensors could enable earlier detection and intervention, reducing sepsis incidence.
Staff support	- The need for additional healthcare providers in pediatric ECMO care due to its complexity and staffing shortages that strain resources.	- This was a frequently mentioned need. - Additional healthcare providers can help to alleviate stress from the high demand in ECMO patient management.

As summarized in Table 3, bleeding was recognized as a prominent issue in ECMO use, particularly for neonates and pediatric patients, as well as patients with open-heart surgery. Some clinicians highlighted the importance of monitoring general trends over time instead of relying on single data points. It was advised by some interviewees that the hemoglobin trend could provide a more comprehensive view of a patient’s bleeding status. One clinician expressed frustration over frequent blood tests (such as PTT) required for ECMO monitoring. It was noted that the lack of automation in this process often disrupted workflow and placed significant strain on the clinical team, underscoring the practical challenges of preventing and managing bleeding complications.

Thrombosis and clotting in ECMO circuits, particularly in MLs, were also significant pain points. One practitioner discussed the challenges of managing clotting in ECMO due to the large variance in patient responses (especially pediatric patients) to anticoagulation therapies. Another professional reaffirmed concerns about clotting, noting that when clots form in the ML, they tend to occur throughout the entire circuit. The preparation needed for an ML swap takes about 15–30 min (or longer), while the actual swap typically takes less than 2 min, when patients would lose ECMO support. One clinician commented that thrombosis was not immediately urgent when the circuit was functioning properly; therefore, regular maintenance and preventative strategies were essential.

Hemolysis and formation of cellular debris in the bloodstream were another major pain point. One clinician expressed concerns about cellular debris accumulating in vital organs, and another emphasized the need for better detection methods to quantify and monitor cellular debris formation. Currently, hemolysis and cellular debris formation in ML can be visually examined using a flashlight, but this method is not always accurate. Emerging techniques include radiographic imaging (x-ray [135] or computed tomography [136]), scanning electron microscopy [137], and low-frequency acoustics [138]. These techniques can be used for post-ECMO support but are not used by clinicians in real-time. One interviewee commented on the importance of quantifying clot formation in ECMO oxygenators. It was suggested that predictive biomarkers may better support healthcare providers in their decision-making process.

For ECMO’s gas exchange performance, some clinicians suggested that real-time monitoring of oxygen levels and pressure within the ECMO circuit could help streamline interventions and reduce the likelihood of membrane lung failure.

Flow and pressure monitoring were also recognized as crucial in managing ECMO systems. One professional stressed the need for more advanced sensors for automatic recording, which could support electronic medical record (EMR) and simplify the documentation and monitoring process. It is important to note that some centers do have access to consoles with advanced sensors, but these devices are costly and thus are limited by the institutional infrastructure and budget. Another clinician pointed out that the time required to monitor patients and assemble teams during critical situations often led to delays in care, reinforcing that quick response times were vital when managing emergencies such as ML failures or acute blood clotting events. While these events may appear suddenly, there may be gradual circuit changes (i.e., the development of a thrombus or decreasing gas efficiency) that may not be detected by routine ECMO monitoring. This emphasizes the need for automated data collection or AI-assisted algorithms to detect emerging issues in the circuit before they escalate into emergencies.

It was reported that ML and cannulas were almost always colonized by biofilms, exposing ECMO patients to infectious complications [139]. Infection, particularly sepsis, remains a serious risk requiring continuous vigilance, a challenge during staff shortages. Staffing and workflow management were among the most frequently cited pain points, with perfusionists and technicians required on call for critical interventions. Several interviewees also raised concerns about stress and burnout among nursing staff given the intensity of ECMO monitoring. Automated, smart monitoring platforms were seen as a means of offloading this burden and improving both outcomes and cost-efficiency.

Some of the key ECMO monitoring parameters mentioned in the literature review section, such as oxygenator blood volume (OXBV) [119], pressure drop across the ML, resistance, and advanced gas exchange metrics were not widely referenced by the interviewees. Topics such as the perceived prevalence of clotting events, device failures, and potential solutions to address the identified challenges were not explored during the interviews, as the primary goal of the customer discovery process was to identify pain points from stakeholders, rather than to validate or discuss technical solutions.

The customer discovery study had some limitations. The geographic distribution of interview participants was concentrated in the northeastern United States, with approximately 58% of interviewees drawn from New York State, primarily urban and academic medical centers in the greater New York City and Long Island region. This pattern was partly attributable to the regional scope of the NSF I-Corps program through which the study was conducted. ECMO monitoring practices, institutional staffing models, resource availability, and receptivity to new technology may vary largely across rural, community-based, or geographically diverse centers outside this corridor, and the identified pain points may not fully represent the broader national landscape. Future customer discovery efforts should intentionally sample from geographically and institutionally diverse ECMO programs to more comprehensively characterize monitoring needs and implementation barriers across varied practice settings.

### Outlook for smart ECMO monitoring

**Figure 1:**
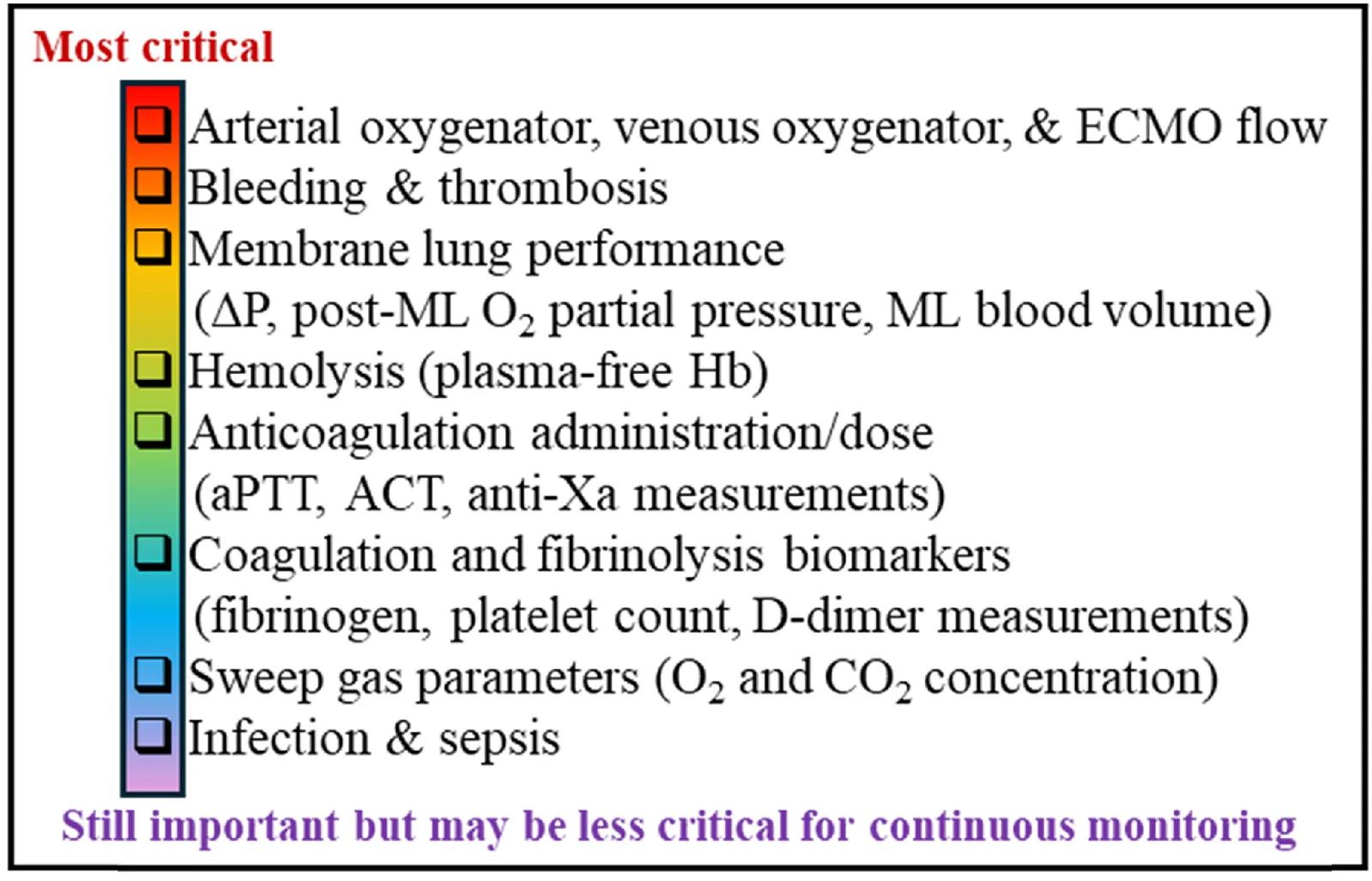
ECMO circuit and patient parameters discussed in the literature review and customer discovery study, in the order of perceived importance for continuous monitoring.

Based on our literature review and customer discovery study, it is not difficult to draw the conclusion that ECMO care and monitoring of the patient and circuit consume significant resources, due to the complex anticoagulation management, continuous and intensive data collection, and frequent treatment adjustments needed to accommodate patients’ dynamic conditions. Figure 1 summarizes all ECMO circuit and patient parameters from both study components, ranked by perceived importance for continuous monitoring. A highly skilled and attentive care team is essential to effectively monitor all these parameters, ensure patient safety, and achieve optimal outcomes in ECMO use.

Alternatively, improvements in ECMO circuit monitoring technology are needed to reduce manual workload and streamline patient care, while minimizing bleeding, clotting, and other adverse events. Navigating the complex, multivariable data stream from ECMO sensors in conjunction with routine clinical data recording in a patient’s chart will play a central role in devising useful ECMO circuit decision support systems. Commercially available platforms have gained traction for high-frequency, integrated data capture (such as Talis Perfusion with +ACG from *Getinge* and *Quantum Informatics* − 24 from *Spectrum Medical*). Concepts such as real-time prognostic models (based on complex data collection and analysis) that can provide advanced clinical decision support for clinicians, digital twins, and “fly by wire” approaches have been proposed and were discussed in a recent review by Pladet et al. [140]. However, if data collected on these platforms cannot be readily shared with the main EMR, health professionals may have limited access to the data.

Early, safe weaning is widely regarded as the best strategy to avoid ECMO complications, but it is not feasible for many patients. Comprehensive, continuous monitoring is therefore essential – both to prevent complications and to inform timely weaning decisions.

A key step toward the future goal of developing a comprehensive monitoring and prognostic system for ECMO care is to gain a better understanding of the complex ECMO-patient data ecosystem and improve access to critical care datasets, especially those including ECMO time-series data. ECMO databases and registries with high-density time series data are not widely available in the US or internationally [1, 141]. So far, ELSO hosts the largest ECMO registry and has a wide range of recorded ECMO cases, variables, and outcomes. However, since ELSO only captures device parameters initially (e.g., flow rate recorded at 4 and 24 h following ECMO initiation), many short-term variations during ECMO operation are not captured. Therefore, the ELSO registry data likely overlooks subtle changes in device measurements that precede acute circuit events. The Medical Information Mart for Intensive Care (MIMIC)-IV is a well-known, massive database covering 65,000 patients admitted to the ICU [142]. But only a small portion of patients [80] have ECMO flow and pressure data sampled, and membrane lung models and membrane lung exchanges are not specified. The Amsterdam University Medical Center’s Database contains patients with ECMO time-series data, but membrane lung exchange is not marked [143]. A comprehensive ECMO registry that includes large numbers of cases and detailed recordings of membrane lung flow rates and pressure gradients, the ML model, system exchange events, oxygen fraction of sweep gas, bleeding and clotting events, hemolysis, etc, will give clinicians and researchers opportunities to collaborate and investigate how each of these parameters impacts the overall patient outcome, which can help to standardize and improve ECMO management. Expanding ECMO registries with compressive time-series data would only be possible with automated (rather than manual) data collection, and data analysis will require machine learning or AI-assisted approaches. Such datasets would support demographic analyses, benchmark identification, and ultimately improved patient outcomes. A current step forward in this direction is the Extracorporeal Life Support Common Data Model (ECLS CDM) [144, 145], which is a community-driven model inspired by the infrastructure of the ELSO Registry, with the goal of supporting ECMO data standardization and sharing of massive ECMO time-series datasets. Therefore, critical parameters used to effectively assess ECMO circuit health must not only be continuously monitored and recorded but also systematically stored in a standardized database. Such a database should be accessible across clinical centers and different user groups to support learning, case studies, and collaborative research. Thus, building a comprehensive ECMO database goes hand in hand with implementing holistic ECMO circuit monitoring.

Another key area to consider is the current lack of automated data transmission and analysis pipelines for ECMO machines and patients. It is necessary to develop a standardized, foundational data pipeline to automatically collect, transmit, and connect detailed machine-level ECMO sensor data to clinical data in a standardized form [144]. Thus, at any given time, clinicians can have a comprehensive view of all important ECMO parameters, from ML flow parameters to blood testing results, without potential human errors or laboratory delays. This will contribute to a new standardized language for ECMO circuit organization and monitoring, in which sensor data, clinical parameters, timestamps, and data formats are consistent between the ECMO console and the electronic medical record system. This can also support machine learning-based algorithm development to identify predictors and early-warning signs as clinical decision support to improve patient outcomes, while significantly reducing staffing burden and workloads.

In summary, this review identified key parameters for ECMO circuit health and examined challenges and opportunities in monitoring, automation, data analysis, and standardization across the literature and clinical perspective. A holistic approach, integrating real-time data analysis and trend analysis, potentially with digital twin technology as a clinical decision support system, represents the future of effective ECMO monitoring.

## Data Availability

No new data were created or analysed in the study.

## Supplementary material

Supplementary document: Customer Discovery Interview Questions Supplementary material of this article is available at https://ject.edpsciences.org/10.1051/ject/2026022/olm.
