# Supplementary material for: Challenges and opportunities in ECMO monitoring: A mixed-methods appraisal
Source: J Extra Corpor Technol. 2026 Sep 15;58(3):280–94. doi: 10.1051/ject/2026022 (PMC13577623; doi:10.1051/ject/2026022)
Supplement: Supplementary file 1 [file ject-58-280-s1.pdf]

Customer Discovery Interview Questions

1. What are the main challenges you face when caring for patients on ECMO, especially related to thrombosis and circuit obstruction?
2. Do you witness any clotting in the system? How long does it take to make critical decisions for the patient?
3. How often do you replace the oxygenator per patient?
4. Can you describe the process and timeline for replacing an oxygenator once a clot is detected?
5. Can you describe your current documentation workflow? (manual vs. EMR)
6. How do you monitor the chatters/indicators for clot formation?
7. Can you describe the current monitoring methods and tools used for patients on ECMO?
8. What types of biometrics and data would you consider most critical for monitoring patients on ECMO?
9. How long does it take for you to evaluate your decisions regarding a patient's condition on ECMO?
10. How long does it take for you to record this data on ECMO?
11. Do they routinely check APTT every 6hrs; how long did this take in your experience?
12. Are there general side effects specific in pediatric/neonatal on long term ECMO?
13. If there was a smart monitoring device for ECMO, would this improve your workflow?
14. If you can improve the ECMO system and process, what would it be?
15. Are there any concerns or reservations you have about implementing a new technology like this in the ECMO workflow, and how could those concerns be addressed?

16. Are there any particular features you would like to see in next-generation ECMO devices?
